# Cholinergic influence on memory stages: A study on scopolamine amnesic mice

**DOI:** 10.4103/0253-7613.56072

**Published:** 2009-08

**Authors:** Rahul Agrawal, Ethika Tyagi, Gunjan Saxena, Chandishwar Nath

**Affiliations:** Division of Pharmacology, Central Drug Research Institute, Lucknow - 226 001, India; 1 Division of Toxicology, Central Drug Research Institute, Lucknow - 226 001, India

**Keywords:** Acquisition, consolidation, passive avoidance, recall, scopolamine

## Abstract

**Objectives:**

The study was planned to determine cholinergic influence on different stages of memory - acquisition, consolidation and recall in scopolamine-induced amnesia (memory impairment) in mice.

**Materials and Methods:**

To study acquision, consolidation and recall stages of memory, we administered scopolamine (0.75, 1.5 and 3 mg/kg ip) 30 minutes and five minutes prior to first trial acquisition and consolidation and 30 minutes prior to second trial recall of passive avoidance (PA) test, respectively, in separate groups. Tacrine (5 mg/kg po) and rivastigmine (5 mg/kg po) were administered one hour prior to first trial in separate groups which received scopolamine (3 mg/kg ip) 30 minutes and five minutes prior to first trial where as the control group received vehicle only.

**Results:**

In the control group, there was a significant (*P* < 0.01) increase in transfer latency time (TLT) in the second trial compared to first indicating successful learning. In scopolamine treated groups, administering scopolamine 30 minutes or five minutes prior to first trial did not show any significant (*P* > 0.05) change in TLT whereas mice treated with scopolamine 30 minutes prior to second trial showed significant (*P* < 0.01) increase in TLT in second trial as compared to the first. Both tacrine and rivastigmine administration in scopolamine treated mice showed significant (*P* < 0.05-0.01) increase in TLT in second trial as compared to first trial while the rivastigmine treated group showed greater percentage retention compared to tacrine treated group.

**Conclusion:**

Results show that acquisition and consolidation are more susceptible to the scopolamine effects than recall. Thus, it may be concluded that cholinergic influence is more on acquisition and consolidation as compared to recall.

## Introduction

Based on experimental and clinical evidences, acetylcholine (ACh) is considered the most important neurotransmitter involved in regulation of cognitive functions.[1,2] Alzheimer's disease (AD) is the most common age related neurodegenerative disorder characterized by cognitive dysfunction with memory impairment and behavioral disturbances.[3,4] Besides the neuropathological hallmarks of the disease, neurofibrillary tangles and neuritic plaques, AD is characterized by a consistent deficit in cholinergic neurotransmission particularly in basal forebrain. The therapeutic strategies to combat miseries of cognitive disorder have been aimed to improve ACh activity. Therefore, the cholinergic receptor agonists (muscarinic and nicotinic) and enhancers of endogenous level of ACh (synthesis promoters and inhibitors of its metabolizing enzyme) have been tried to treat senile dementia of Alzheimer type. Among the various approaches attempted to increase cholinergic activity, the inhibition of acetylcholiesterase (AChE) is the most successful one.[5] Cholinesterase inhibitors are the only class of compounds consistently proven to be efficacious in treating the cognitive and functional symptoms of AD.[6]

Blockade of the central muscarinic acetylcholine receptor disrupts learning and memory functions in animals as well as human beings. Anticholinergic drugs (muscarinic blocker) such as scopolamine have been in use as potent amnesic agents. Scopolamine-induced cognitive deficit in young volunteers is similar to that occurring in senile, demented subjects when tested on same clinical battery. One such deficit in young subjects was loss of memory of recent (but not immediate) events.[7] Subsequently, several studies reported that the cholinergic receptor antagonists atropine and scopolamine administered before training impaired performance of rats on various types of tasks.[8,9] Scopolamine model of amnesia is very commonly used in screening of memory enhancing drugs.[10]

The drugs administered before behavioral testing can influence sensory processing, attention and motivation as well as brain processes underlying the storage of new information.[11] The use of post training drug administration provided a method of investigating drug effects on memory consolidation without the confounding effects of drug on pain threshold or motor activity during the acquisition phase.[12] These studies highlight the importance of administration of drugs in relation to stages of memory process. There are three basic activities associated with memory process - acquisition, consolidation and recall.[13] If the effect of drugs varies with time of administration in an experimental procedure of learning, the question which arises is - does cholinergic mechanism differ in stages of memory? Therefore, it was planned to investigate the role of cholinergic influence on different stages of learning and memory process by studying the effect of cholinergic antagonist (scopolamine) and enhancers (tacrine and rivastigmine) in PA.

## Materials and Methods

### Animals

The experiments were carried out on adult male Swiss albino mice weighing 20-25 gm. The animals were kept in polyacrylic cage and maintained under standard housing conditions (room temperature 24-27°C and humidity 60-65%) with 12 hour light and dark cycle. There were five animals in each group. The food in the form of dry pellets and water were available *ad libitum*. The animals were procured from the Laboratory Animal Services Division of Central Drug Research Institute, Lucknow, India. Experiments were performed as per internationally followed ethical standards, after clearance from ethics committee of Central Drug Research Institute and Committee for the Purpose of Control and Supervision of Experiments on Animals (CPCSEA), Government of India, on animal experimentation.

### Passive avoidance test

The mice were subjected to single trial PA test as described by Das *et al*.[14] To begin with, one animal is placed in the lit compartment of a computerized shuttle box (Columbus Instruments, Ohio, USA) provided with a software program PACS 30. An automated guillotine door isolated the compartment lit at intensity of 8 (scale of 0 - off and 10- brightest provided in the PACS 30 software) from the dark compartment. After the acclimatization period of 30 seconds the guillotine door automatically opens and the animal is subjected to a trial of 270 seconds. An entry into the dark compartment automatically shuts the door and the subject is punished with a single low intensity foot shock (0.5 mA; five seconds). Infrared sensors monitor the transfer from one compartment to another, which is recorded as TLT in seconds. The first trial is for acquisition and the retention is tested by a second trial given 24 hours after the first trial. The shock is not delivered in the second trial. The criterion for learning is taken as significant increase in the TLT on second trial (retention trial) as compared to first trial (acquisition trial). The comparison between the effects of the two groups on PA is done on the basis of percentage retention i.e. percentage increase in TLT of second trial from TLT of first trial.

### Drug administration

Scopolamine (0.75, 1.5 and 3 mg/kg) is administered intraperitoneally, at different time points to study its effect on acquisition, consolidation and recall stages of memory in PA test as follows:

Group I - Scopolamine treatment groups:

- The doses 0.75, 1.5 and 3 mg/kg of scopolamine are administered intraperitoneally, in separate groups (n = 5), 30 minutes prior to first trial for acquisition.
- The doses 0.75, 1.5 and 3 mg/kg of scopolamine are administered intraperitoneally, in separate groups (n = 5), 5 min prior to first trial for consolidation.
- The doses 0.75, 1.5 and 3 mg/kg of scopolamine are administered intraperitoneally, in separate groups (n = 5), 30 minutes prior to second trial for recall.

Group II - *Drug treatment groups:*

(i) Vehicle, (ii) tacrine (5 mg/kg po) or (iii) rivastigmine (5 mg / kg po) is administered one hr prior to first trial[15,16] in separate groups (ns equal to5) treated with scopolamine (3 mg / kg ip) 30 minutes and five minutes prior to first trial to observe the effect on acquisition and consolidation respectively.

For each group there is a non scopolamine control group (n = 5) that received only normal saline (10 ml/kg ip). All the drugs are dissolved in normal saline.

### Statistical analysis

The statistical analysis was performed by student's (paired) ‘*t*’ test and one way analysis of variance (ANOVA) followed by Newman Keuls test. In the analysis, any values of *P* < 0.05 were considered statistically significant.

## Results

### Effect of scopolamine on different stages of Passive Avoidance memory functions

**Figure 1:**
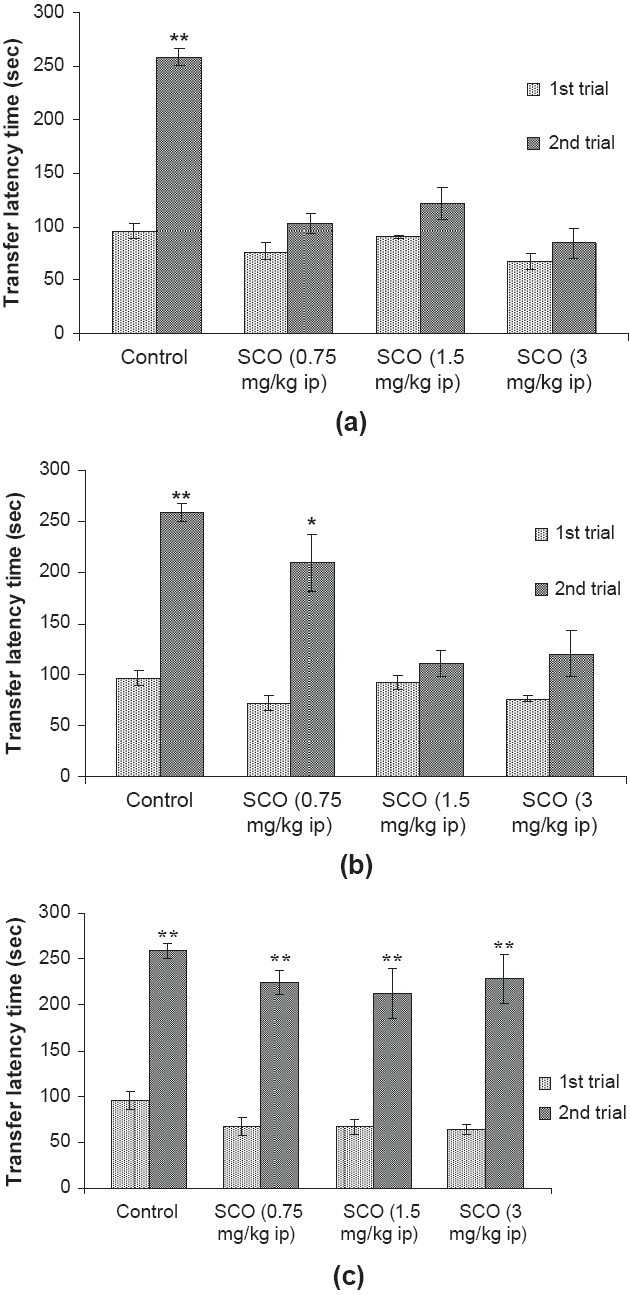
Effect of scopolamine (SCO; 0.75, 1.5 and 3 mg/kg ip) on transfer latency time (TLT) in the PA test to observe the effect on (a) acquisition, administered 30 minutes prior to first trial, (b) consolidation, administered 5 min prior to 1st trial and (c) recall, administered 30 minutes prior to second trial. The maximal time of latency was set at 270s (cutoff time). Values are expressed as mean ± SEM; n = five. *P < 0.05, **P < 0.01 significant increase in TLT on second trial as compared to their respective first trial; student's (paired) ‘t’ test.

The TLT was found significantly increased [*P* < 0.01, *df* = four] on the second trial as compared to first trial in control group, indicating that animals had acquired the task. Mice treated with scopolamine at the doses of 0.75, 1.5 and 3 mg/kg ip 30 minutes prior to first trial for acquisition did not show any significant change [*P* > 0.05, *df* = four] in transfer latency time on second trial as compared to first trial. There was no significant difference [*F*(3, 16) = 2.827, *P* > 0.05] found in TLT in the first trial of different groups [Figure 1a].

When scopolamine was administered five minutes prior to first trial for consolidation at the dose of 0.75 mg/kg ip, the significant increase [*P* < 0.05, *df* = four] was found in transfer latency time on second trial as compared to first trial. However, scopolamine at the doses of 1.5 and 3 mg/ kg ip did not show any significant change [*P* > 0.05, *d* f = four] in transfer latency time on second trial as compared to first trial. There was no significant difference [*F*(3, 16) = 1.971, *P* > 0.05] found in transfer latency time among the first trial of different groups [Figure 1b].

Mice treated with scopolamine at the doses of 0.75, 1.5 and 3 mg/kg ip 30 minutes prior to second trial for recalling process showed significant increase [*P* < 0.01, *df* = four] in TLT on second trial as compared to first trial. There was no significant difference [*F* (3, 16) = 2.963, *P >* 0.05] found in TLT among the first trial of different groups [Figure 1c].

### Effect of tacrine and rivastigmine on scopolamine-induced impaired acquisition of Passive Avoidance memory

**Figure 2:**
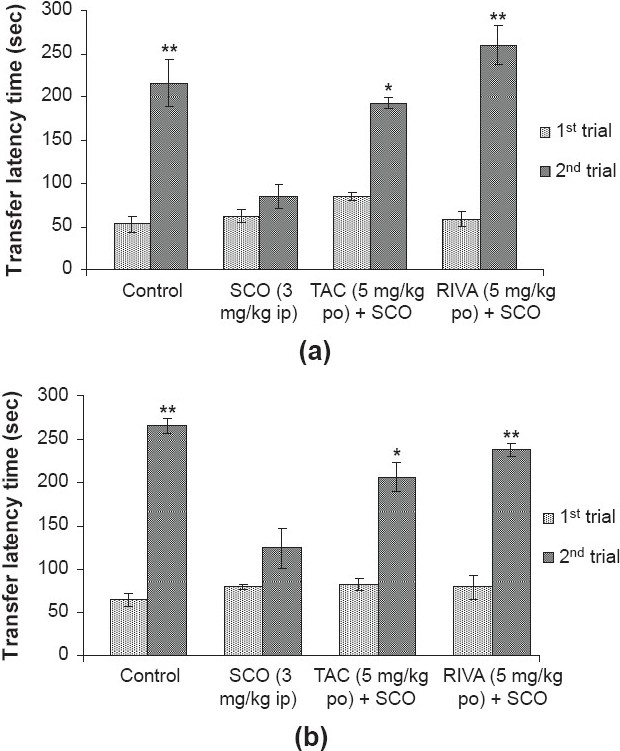
Effect of tacrine (TAC; 5 mg/kg po) and rivastigmine (RIVA; 5 mg/kg po) on transfer latency time (TLT) in the PA test to observe the effect on scopolamine (SCO; 3 mg/kg ip) induced impaired (a) acquisition, administered 30 minutes prior to first trial and (b) consolidation, administered five minutes prior to first trial. The maximal time of latency was set at 270s (cutoff time). Values are expressed as mean ± SEM; n = five. *P < 0.05, **P < 0.01 significant increase in TLT on second trial as compared to their respective first trial; student's (paired) ‘t’ test

There was a significant increase [*P* < 0.01, *df* = four] in TLT on second trial as compared to first trial in saline treated (30 minutes prior to first trial) control group indicating that animals had acquired the task, whereas mice treated with scopolamine (3 mg/kg ip), 30 minutes prior to first trial, did not show any significant change [*P* > 0.05, *df* = four] in TLT time on second trial as compared to first trial indicating no acquisition. The tacrine (5 mg/kg po) and rivastigmine (5 mg/kg po) administration (one hour prior to first trial) in scopolamine treated group showed significant increase [*P <* 0.05, *df* = four; *P* < 0.01, *df* = four] in TLT on second trial as compared to first trial. There was no significant difference [*F*(3, 16) = 2.856, *P* > 0.05] in TLT in the first trial of different groups [Figure 2a].

**Figure 3:**
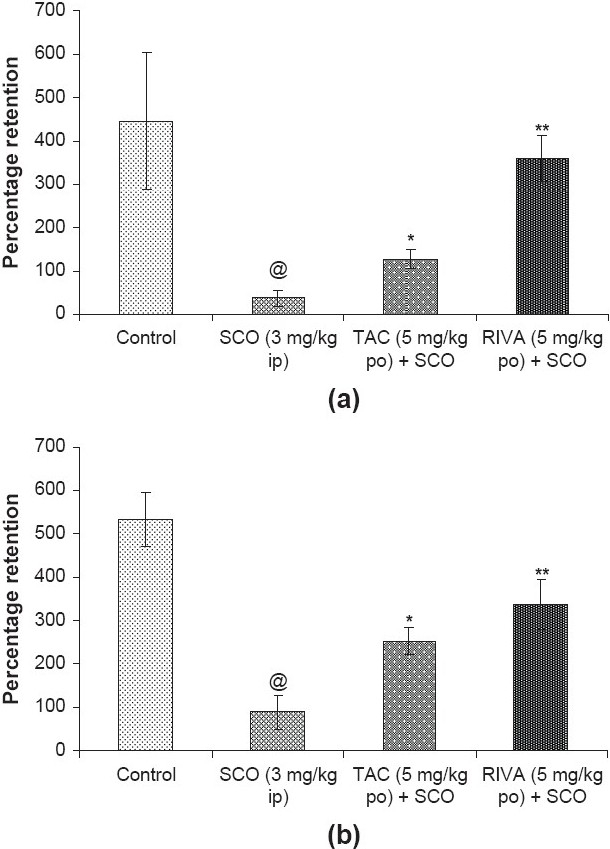
Percentage retention in control, scopolamine (SCO; 3 mg/kg ip), tacrine (TAC; 5 mg/kg po) plus scopolamine (SCO) and rivastigmine (RIVA; 5 mg/kg po) plus scopolamine (SCO) treated groups for (a) acquisition and (b) consolidation. @*P* < 0.05, significantly different from control; *P < 0.05, **P < 0.01, significantly different from scopolamine treated group; ANOVA (one-way) followed by Newman–Keuls test

However, rivastigmine (5 mg/kg po) treated group showed greater percentage retention as compared to the tacrine (5 mg/kg po) treated group. The percentage retention in control, scopolamine, tacrine and rivastigmine treated groups were 445.5, 37.72, 126.9 and 358.9 % respectively [Figure 3a].

### Effect of tacrine and rivastigmine on scopolamine-induced impaired consolidation of Passive Avoidance memory

There was a significant increase [*P* < 0.01, *df* = four] in TLT on second trial as compared to first trial in the saline treated (five minutes prior to first trial) control group indicating that animals had acquired the task. However, mice treated with scopolamine (3 mg/kg ip) five minutes prior to first trial did not show any significant change [*P* > 0.05, *df* = four] in TLT on second trial as compared to first indicating impaired consolidation. The tacrine (5 mg/kg po) and rivastigmine (five mg/kg po) administration (one hour prior to first trial) in scopolamine treated mice showed significant increase [*P* < 0.05, df = four; *P* < 0.01, df = four] in TLT on second trial as compared to first trial respectively. There was no significant TLT difference [*F*(3, 16) = 1.248, *P*≥0.05] in the first trial of different groups [Figure 2b].

However, the rivastigmine (five mg/kg po) treated group showed greater degree of percentage retention as compared to tacrine (five mg/kg po). The percentage retention in control, scopolamine, tacrine and rivastigmine treated groups were 331.2, 54.54, 157.4 and 209.8% respectively [Figure 3b].

## Discussion

The importance of central cholinergic system in memory function is well established. The present study provides information on the involvement of cholinergic system on the different stages of memory process. The stages considered as crucial for successful memory are acquisition, consolidation and recall of learned task.[13] Acquisition is the step during which the animal learns a task, consolidation is the process during which the memory is stabilized and recall is bringing back of the learned task. The PA test was employed in the study to assess the stages of memory function. In the PA test, mice acquire the information that moving to a dark chamber results in delivery of electric shock in the first trial. This acquired information delays the entry of mice into the dark chamber to avoid the shock (PA) during second trial after 24 hours. This protocol of PA test demonstrates that the information acquired in the first trial (acquisition trial) is consolidated within 24 hours and successfully recalled during second trial (retention trial).

The cholinergic influence on stages of memory in PA test was determined by investigating the effects of cholinergic muscarinic antagonist scopolamine and its interaction with cholinergic enhancers – anticholinesterase drugs tacrine and rivastigmine. Scopolamine was administered in three different dose regimes at different time points in relation to first and second trials of PA test. As discussed earlier, first trial and second trials are linked with acquisition and recall respectively and the time after first trial represents the phase of consolidation. Therefore, scopolamine was administered 30 minutes prior to first trial to affect the acquisition. In another group, scopolamine was administered five minutes prior to first trial. In this group due to very short interval between scopolamine administration and first trial information can be acquired but consolidation may be affected by scopolamine. Moreover, we did not prefer administration of scopolamine just after first trial to avoid any effect of animal handling on acquired information. It is possible that immediate noxious stimulation due to insertion of needle during administration of scopolamine may interfere with noxious sensation of shock perceived by animal during PA. We employed another regime where scopolamine was administered 30 minutes prior to second trial so that acquisition and consolidation of information (i.e. shock in dark chamber) could be spared but only recall of it should take place under the effect of scopolamine in second trial. It was observed that memory impairment i.e. no significant increase in TLT on second trial, occurred only in first two regimes of scopolamine, which affects acquisition and consolidation. In the other set up where only recall could be affected memory remained intact as shown by significant increase in TLT on second trial.

The most important finding of this study is that acquisition and consolidation process of learned task, rather than recall, is more susceptible to inhibition of cholinergic influence. Das *et al.*[17] made similar observations in the conditioning learning test which showed that systemic administration of scopolamine impaired acquisition and consolidation phases without affecting recall in active avoidance. Gene and David[18] showed that the intrahippocampal scopolamine caused deficits in acquisition and consolidation of contextual fear conditioning. Thus, observations with scopolamine indicate significant role of cholinergic mechanism in acquisition and consolidation but not in recall.

For further confirmation of cholinergic involvement in stages of memory, the effect of cholinergic enhancers – tacrine and rivastigmine on scopolamine-induced impairment in acquisition and consolidation was studied. Tacrine and rivastigmine are clinically used antidementia drugs and increase level of acetylcholine by inhibition of cholinesterase.[19] Pretreatment with tacrine or rivastigmine prevented the inhibitory effect of scopolamine on acquisition and consolidation process of learned task. However, the rivastigmine treated group showed greater percentage retention as compared to tacrine treated group, which might be due to more selective inhibition of acetylcholinesterase in hippocampus by rivastigmine as compared to tacrine.[12] Antagonism of scopolamine-induced deficits in PA learning by anticholinesterase drugs shows the dominance of cholinergic influence during acquisition and consolidation phases in memory functions. Preferential influence of cholinergic system on acquisition and consolidation as observed in the study correlates with the impairment in acquisition and consolidation of recent events and information without affecting past memory in patients of AD.[20]

The preferential effect of scopolamine on acquisition and consolidation of memory in the learning process suggests that the time of administration of scopolamine in relation to stage of learning task is an important factor for producing amnesia in an experimental animal. Thus, it may be concluded from the effects of scopolamine and anticholinesterase drugs found in the study, that cholinergic system influences acquisition and consolidation rather than recall.
